# Cell Death Effects of the Phthalate 2-Ethyl-1-Hexanol on Human Linfoblast Cells

**DOI:** 10.4236/ojapo.2019.81001

**Published:** 2019-01-31

**Authors:** Karoline Rios, Christian Vélez, Beatriz Zayas

**Affiliations:** School of Science and Technology and Environment, Universidad Ana G. Méndez, San Juan, Puerto Rico

**Keywords:** Phthalates, 2-Ethyl-1-Hexanol, Diethylhexyl Phthalate, Autophagy, Apoptosis, Lymphoblast, Caspase

## Abstract

Phthalates have been used in a wide variety of consumer goods. Their versatility as plasticizers has translated into worldwide use in a vast array of consumer products. These compounds can leach into matrices, such as food and liquids that can be routed for human exposure. One of the most used phthalates is Diethylhexyl phthalate (DEHP). Diethylhexyl phthalate and its metabolite 2-ethyl-1-hexanol (2-EH) have demonstrated biological effects which merit further evaluation. In this work, we expand on our previous work with DEHP and screen the 2-EH metabolite for different cell death endpoints such as growth inhibition, apoptosis, autophagy, caspase activation, DNA fragmentation, and cell cycle arrest using fluorophores and the NC3000 instrument. Significant results (*p* < 0.05) revealed higher toxicity for the 2-EH metabolite when compared to DEHP. Also, 2-EH presented apoptosis induction with characteristic hallmarks, such as loss of mitochondrial membrane potential, caspase activation, DNA fragmentation and cell cycle arrest at the S phase. In addition, the presence of autophagosome was detected through L3CB protein staining. We conclude that 2-EH presents differences in cell death endpoints that interestingly differ from the DEHP parent compound. Further studies are needed to establish the molecular pathways responsible for the observed effects.

## 1. Introduction

Modern manufacturing practices have relied on plastics to improve the physical properties of consumer goods. In turn, plastic manufacturing technologies have evolved to modify the characteristics of their products. Phthalates are widely used as plasticizers to increase the flexibility and durability of plastic products. Since the 1920s with the introduction of Diethylhexyl phthalate (DEHP) phtalate ester, compounds have been used extensively in different types of products such as building materials, flooring, piping, food packaging, and medical devices, among others [1] [2] [3]. Exposure to phthalates can occur from food, water air, fabrics, dust, medications, cosmetics, and any plastic-made products. Also, the preparation of food or drink, using microwaves, can promote the leaching of phthalates from containers [4].

**Figure 1.**
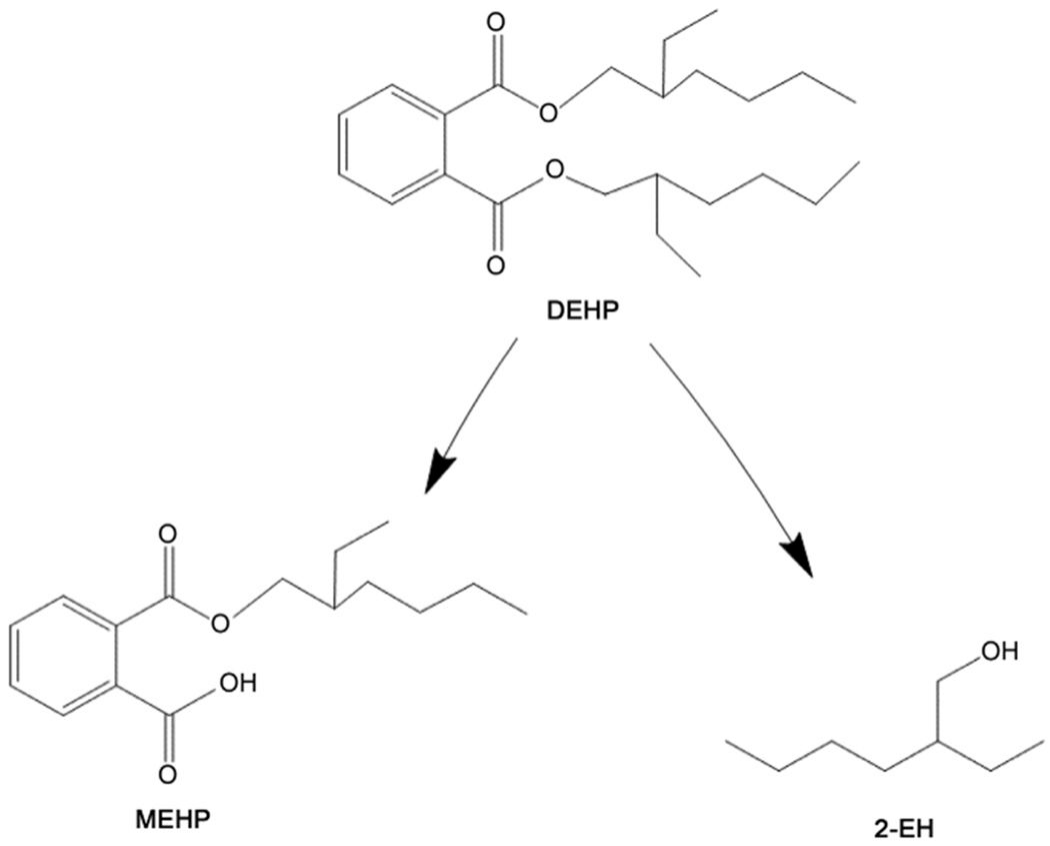
DEHP and its metabolites. MEHP and 2-EH are the principal metabolites from DEHP.

Diethylhexyl phthalate is a common phthalate historically used for imparting flexibility to plastic products including medical devices, such as catheters, blood, and IV bags, among others [5]. The high use of this compound has led to it being detected in air, water, soil and in dust particles [6] and might be persistent in the environment due to a relative low biodegradability [7]. The degradation of DEHP can produce the 2-ethyl-1-hexanol (2-EH) metabolite (Figure 1). This compound can be generated either after ingestion of DEHP and enzymatic degradation into mono-(2-ethylhexyl) phthalate (MEHP) and finally 2-EH [8]. Other sources of 2-EH can include some microorganisms that can degrade diester plasticizers [9].

Our previous work [10] established the toxicity and apoptosis induction of both DEHP and MEHP on human lymphoblast cells. Various cell death hallmarks were documented including mitochondrial membrane permeabilization, generation of reactive oxygen species (ROS), and caspase activation. This work aims to build our knowledge of 2-EH and further establish a comparison of the biological effects among DEHP and 2-EH.

## 2. Materials and Methods

### 2.1. Reagents

2-EH and DEHP were purchased from Sigma Aldrich (St. Louis, MO) and diluted with dimethyl sulfoxide (DMSO) 1% for the working solutions (0.1% final DMSO concentration to cells) which were kept in sterile conditions and refrigerated until utilized. Positive controls: DEHP, camptothecin, and chloroquine for autophagy assays were purchased from Sigma Aldrich. DMSO 0.1% and culture media were used as a vehicle negative control.

### 2.2. Cell Culture

The human lymphoblast cell line TK6 was obtained from the American Type Culture Collection (Manassas, VA. ATCC CRL-8015). Cell cultures were maintained in RPMI 1640 culture media with 10% fetal bovine serum (ATCC). Cells were maintained at 37°C with 5% CO_2_.

### 2.3. Growth Inhibition 50% Determination (GI50)

TK6 Cells were seeded (30,000 cells/well) on fluorometry compatible 96 well plates (NUNC, Rochester, NY) then exposed to the experimental compounds at ten different doses (in triplicates) for 24 hours. After the incubation period, the Prestoblue cell viability reagent (Invitrogen, Carlsbad, California) was added to all wells. This reagent is reduced by metabolically active cells to its fluorescent form and the fluorescence produced by the reduced form is analyzed using a Fluorostar Optima fluorescence reader (BMG lab tech, Cary, NC) using the 485/520 nm filters. Results were analyzed using the MARS data analysis software (BMG). Data with a coefficient of determination (*R*) lower than 98 was not considered. This growth inhibition dose was used in all subsequent experiments.

### 2.4. Annexin V

The annexin V assay is a common assay used in the detection of apoptosis. This assay measures the migration phosphatidylserine (PS) to the exterior surface of cells, a common event in apoptotic cells [11]. Approximately 3.0 × 10^6^ cells were treated for 24 hours with the 24-hour GI_50_ dose for each test compound and the controls camptothecin 10 μM, and vehicle (DMSO). After the exposure period, cells were stained with annexin V conjugate, and propidium iodide (Biotium, Hayward, CA). These stained cells are then analyzed using the Nucleo Counter NC3000 system annexin V assay (Chemometec, Allerød, Denmark). Fluorescence of cells is detected and categorized on fluorescence emitted. A one-way ANOVA with post hoc Tukey test was also performed for all cell death assays (GraphPad Prism v.6).

### 2.5. Mitochondrial Membrane Permeabilization

Mitochondrial membrane permeabilization (MMP) is a key event in the apoptotic process [12]. A total of 1 × 10^6^ cells were exposed to the test compounds and the controls at their GI_50_ doses. Cells suspensions were stained with 200 μg/ml of 5,5’,6,6’-tetrachloro-1,1’,3,3’-tetraethyl benzimidazolo carbocyanine iodide (JC-1). Fluorescence was analyzed using a Fluorostar Optima fluorescence reader (BMG lab tech, Cary, NC) using the 485/520 nm filters. Results were analyzed using the fluorometer’s MARS data analysis software (BMG).

### 2.6. Caspase Activity

Caspase activation has been used as a key endpoint to identify cell death via apoptosis. In brief, apoptosis activation via caspases which can be grouped into two major categories; initiators and effectors. We have selected as endpoints Caspases 3 and 7 as the effectors, and Caspases 8 and 9 as the initiators. Once this signaling is activated, the cell proceeds with activating its enzymatic degradation [13]. Here, cell cultures were exposed to the test compounds as described previously and stained with the Fluorescent Labeled Inhibitors of Caspases (FLICA) purchased from (Immunochemistry Technologies, Bloomington Min.). This compound binds to the active caspase enzymes and emits green fluorescence at 488 - 492 nm peaking at 515 - 535 nm. Higher fluorescence will represent a higher number of cells with active caspase 3 & 7.

### 2.7. DNA Fragmentation

DNA Fragmentation is another common endpoint used to measure apoptotic responses on cells [14]. This event mediated by nucleases generates small fragments of DNA, which can be quantified by examination of DNA content and examining cells containing less than 1 DNA equivalent. The NC3000 assay for DNA fragmentation was used and is based on the removal of small DNA fragments and the retention of higher weight fragments stained with the DNA marker 4’,6-diamidino-2-phenylindole (DAPI). Cells were fixed with 70%, ethanol then stained with 1 μg/ml DAPI, and analyzed using the NC3000 instrument which measures DAPI fluorescence intensity measuring and quantifying high molecular weight DNA fragments.

### 2.8. Cell Cycle

Changes in the cell cycle can provide useful insights into drug effects. Some compounds may cause cells to become arrested in a particular stage [15]. To study 2-EH effects, the NC3000 fixed cell cycle assay was performed. This DAPI based assay uses ethanol-fixed cells then stained with 1 μg/ml DAPI and analyzed to measure by fluorescence DNA content and determination of cell cycle stage.

### 2.9. Autophagy

Autophagy is a key cellular process for maintaining homeostasis, and involves the degradation of subcellular components [16] [17]. During this process, cellular components reach lysosomes for degradation [18]. It has been documented that phthalates can cause autophagic cell death with mitochondrial effects [19]. The Premo autophagy sensor kit was purchased from Thermo Fisher (Waltham, MA). This probe binds to the LC3B protein, which is used to detect autophagic membranes. Approximately, 4.0 × 10^4^ cells were treated using the Premo kit following manufacturer’s specifications and plated in 96 well plates then is analyzed using a Fluorostar Optima fluorescence reader (BMG lab tech, Cary, NC) using the 485/520 nm filter.

### 2.10. Cathepsin B

Cathepsins are a group of proteases present in lysosomes and present during autophagy [20]. These enzymes degrade intracellular and extracellular materials into their basic forms reusable by the cells [21]. The green cathepsin B assay (Immunochemistry Technologies, Bloomington MN) was used to detect cells with elevated cathepsin B levels. Cells were stained following the manufacturer’s specifications and analyzed using a Fluorostar Optima fluorescence reader (BMG lab tech, Cary, NC) using the 485/520 nm filter.

## 3. Results

### 3.1. Growth Inhibition 50% Determination (GI_50_)

**Figure 2.**
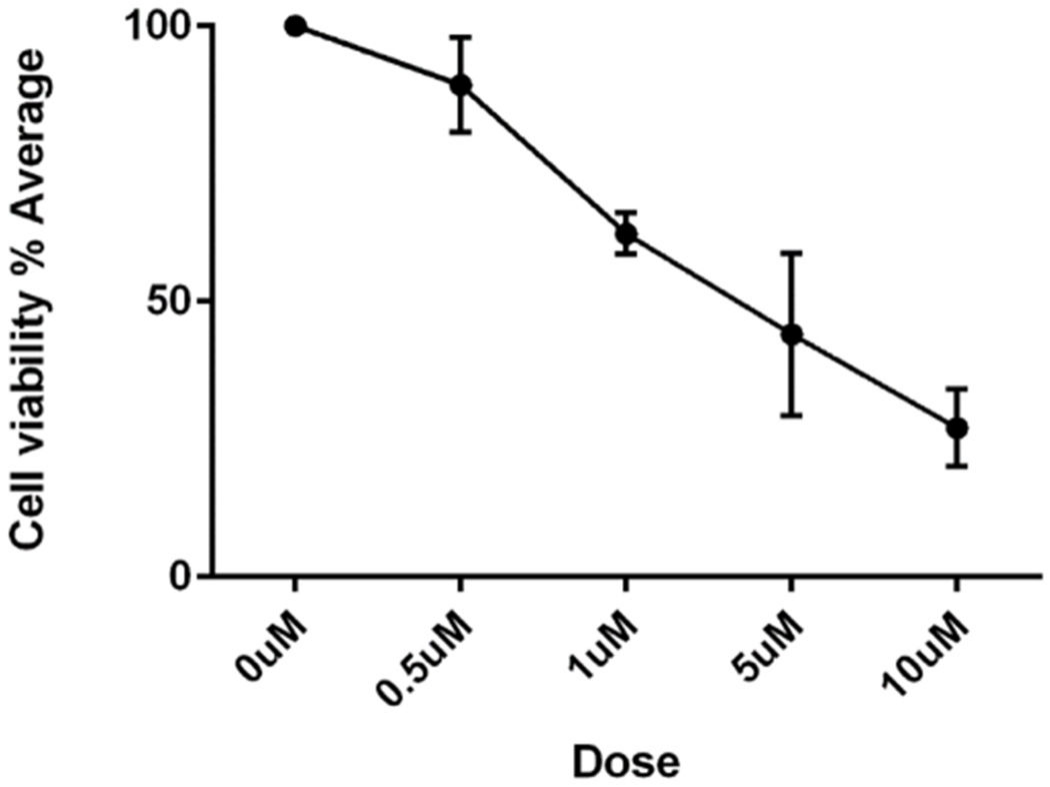
24 exposure dose response 2-EH. This compound presented average toxicity of 4.4 μM. Cell death effects present dose-dependent toxicity. Figure 2 presents results from 5 doses with average data from 3 experiments; error bars indicate standard deviation.

Growth inhibition 50% (GI_50_) is commonly used as a measure of the inhibition of 50% of exposed cells to a substance. The growth inhibition dose of 4.4 μM for 2-EH and 76 μM for DEHP on TK6 cells were determined after a 24-hour exposure (Table 1). The measurement of fluorescence corresponding to viable metabolically active cells was measured at 24 hours. The 2-EH compound presented a dose-dependent inhibition of all tested doses (Figure 2).

### 3.2. Annexin V

**Figure 3.**
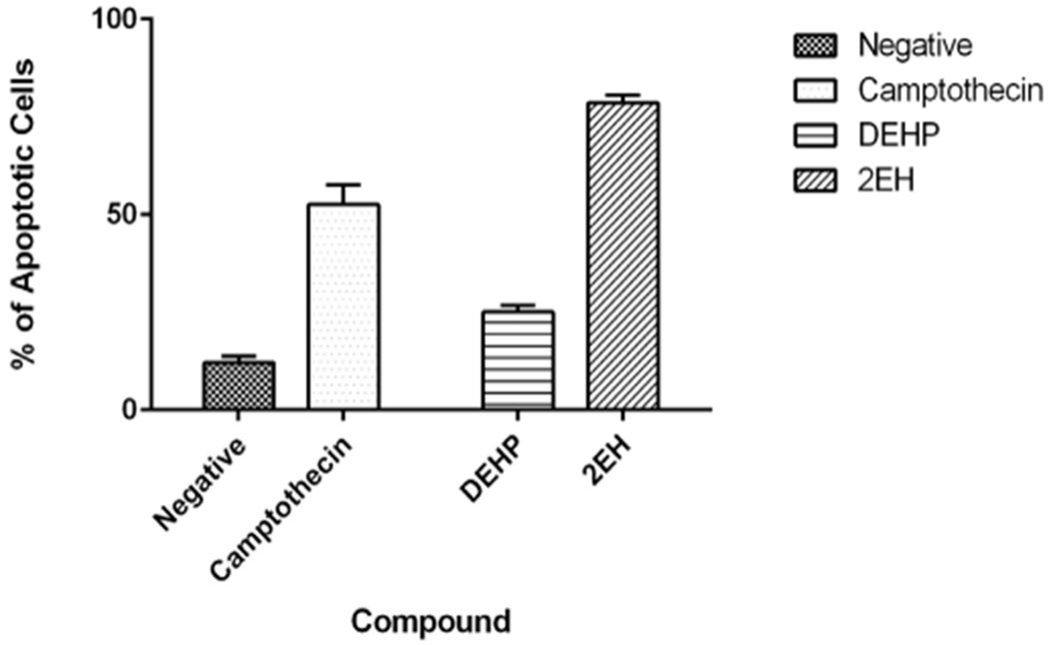
Annexin V apoptosis assay. After a 24-hour exposure period our results reveal statistically significant (*p* < 0.05) apoptosis induction by all the test compounds. DEHP was the lowest inducer of apoptosis but still presented significant induction when compared to the negative vehicle control.

Results for annexin V assay revealed statistically significant (*p* < 0.05) induction of apoptosis when compared to the negative vehicle control (Figure 3). The negative control presented an average of 12% apoptotic cells whereas the positive camptothecin control presented a 52.6% average. The experimental compounds present late apoptotic events with 25% for the parent DEHP and 78.6% for the 2-EH samples.

### 3.3. Mitochondrial Membrane Permeabilization

**Figure 4.**
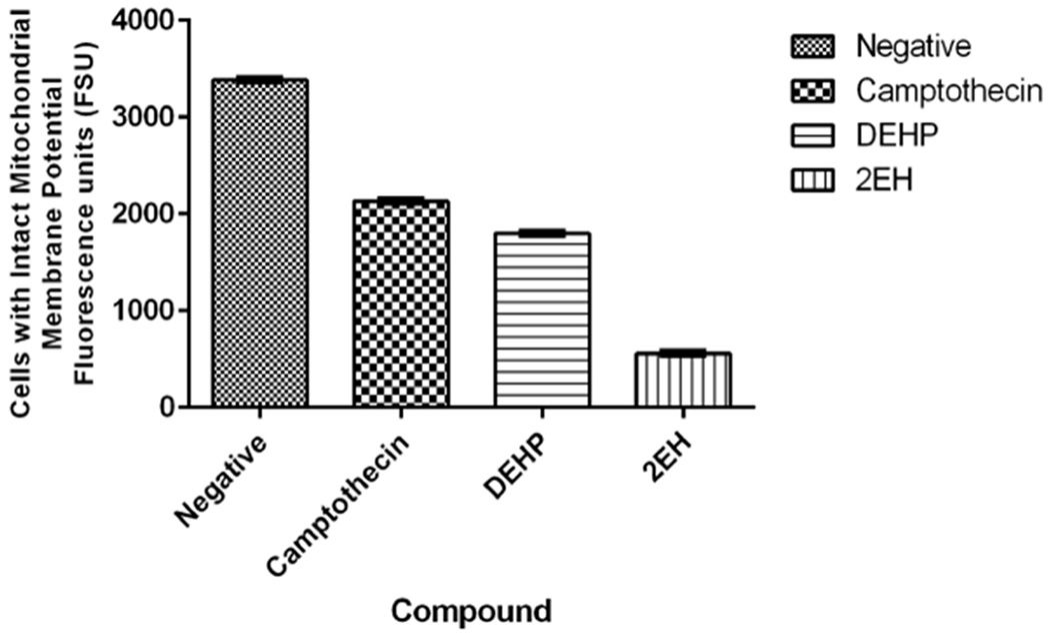
Mitochondrial membrane potential. Negative samples reflect an average of 3380 Fluorescence Standard Units (FSU) while the camptothecin control presented an average of 2130 FSU. The phthalate samples reflect values of 1797 FSU for DEHP and 556 FSU for 2-EH. The 2-EH sample presents the least number of cells with intact mitochondrial membrane potential.

Mitochondrial health was assessed after exposure to the phthalates and the controls. Statistically significant (*p* < 0.05) differences were detected on all test compounds when comparing to the negative control (Figure 4). Mitochondrial membrane health was assessed by measuring the green fluorescence (Fluorescence Standard Units or FSU) of JC-1 stained cells. Cells with an intact mitochondrial membrane will emit orange fluorescence, whereas cells with compromised membranes will fluoresce green. Negative samples reflected an average of 3380 FSU while the positive camptothecin control presented an average of 2130 FSU. The phthalate samples reflected values of 1797 FSU for DEHP and 556 FSU for 2-EH. The 2-EH presented the least number of cells with intact mitochondrial membrane potential (ΔΨ_*m*_).

### 3.4. DNA Fragmentation

**Figure 5.**
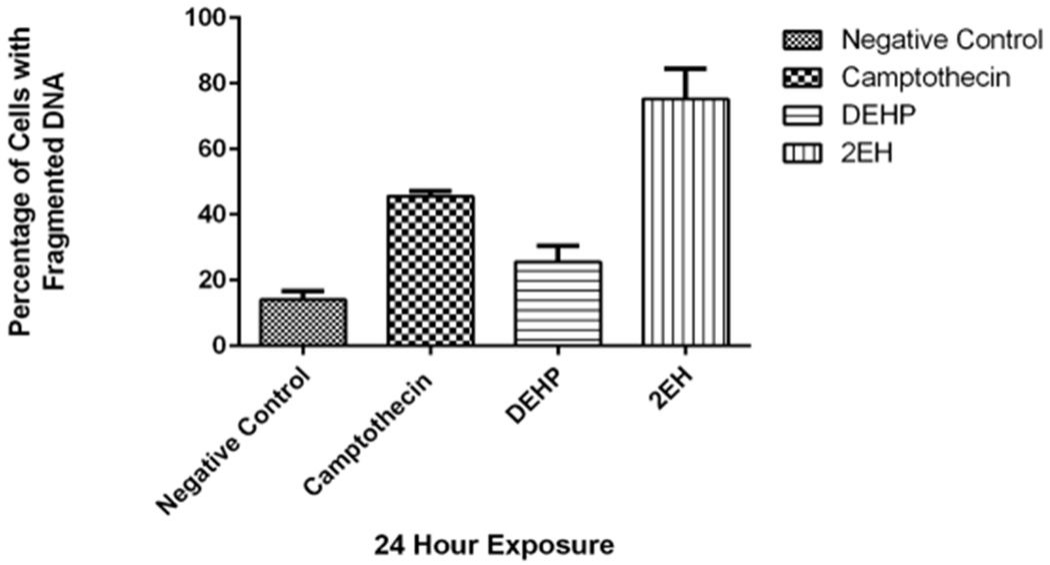
Cells with fragmented DNA. the negative control presented an average of 14.2% and 45.6% for the positive camptothecin control. DEHP presented an average of 25.6% and 2-EH (75.3%) presented a majority of cells with fragmented DNA. DEHP value was not statistically significant (*p* < 0.05) when compared to the negative control.

Figure 5 presents our DNA fragmentation results which presented an average of 14.2% for the negative control and 45.6% for the positive control. DEHP presented an average of 25.6% which was not statistically significant when compared to the negative control and 2-EH (75.3%) presented a majority of cells with fragmented DNA indicating cells undergoing the cell death process.

### 3.5. Caspase Activity

#### 3.5.1. Caspase 3

**Figure 6.**
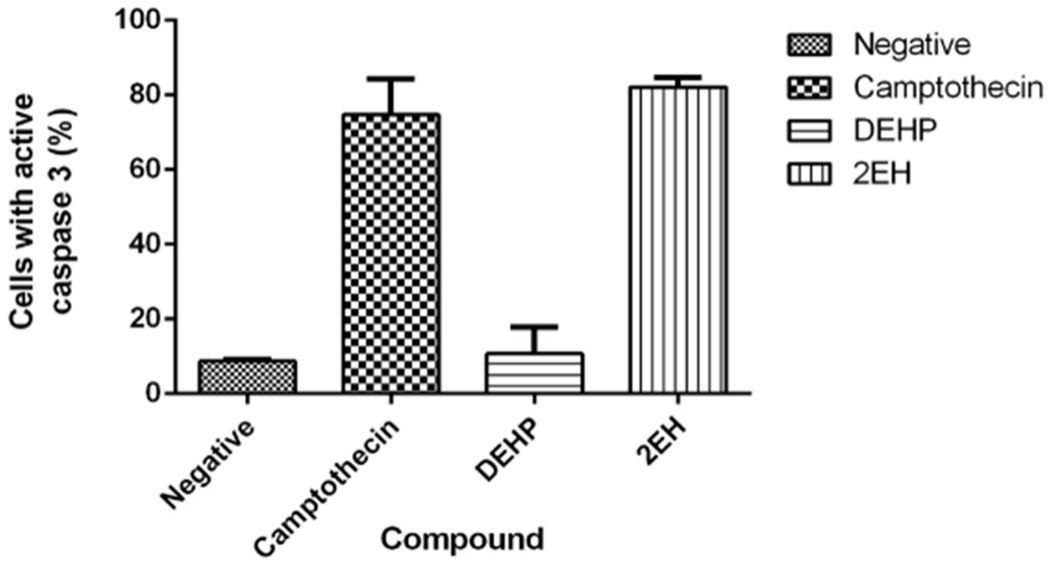
Caspase 3 activation. Only camptothecin and the 2EH samples presented caspase 3 activation. DEHP presented 10.6% of cells with active caspase 3 comparable to the negative control (8.6%). Only the positive control camptothecin (74.6%) and 2-EH (82.0%) presented statistically significant (*p* < 0.05) caspase 3 activation.

Activation of effector caspase 3 was detected in the camptothecin positive control and 2-EH samples. Camptothecin samples presented an average of (74.6%) and the 2-EH samples an average of 82.0%. both samples presented statistically significant (*p* < 0.05) caspase 3 activation (Figure 6). DEHP samples and the negative control did not activate caspases in a significant manner with 10.6% and 8.6% respectively.

#### 3.5.2. Caspase 8

**Figure 7.**
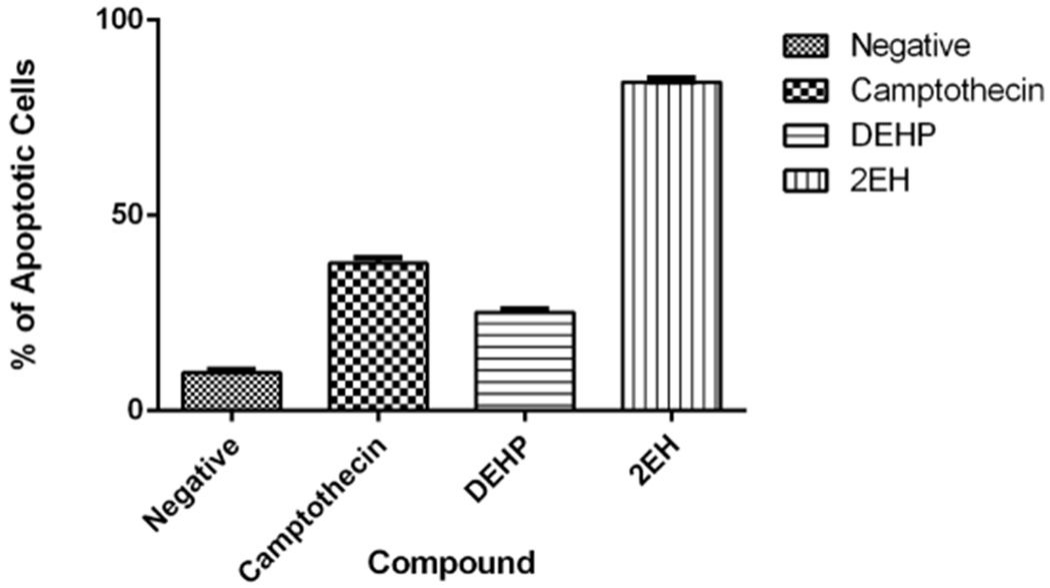
Caspase 8 activation. Negative vehicle sample averaged 9.6% of caspase 8 active cells with the positive camptothecin averaging 37.6% activation. DEHP samples presented 25.0% and 84.0% for 2-EH. Both phthalate compounds activated caspase 8.

Messenger caspase 8 activation was detected in all treated samples in a statistically significant (*p* < 0.05) manner. Negative samples averaged 9.6% of caspase 8 active cells and the camptothecin positive control average 37.6% activation. Phthalate samples presented 25.0% and 84.0% for DEHP and 2-EH respectively (Figure 7). Only the negative sample did not activate caspase 8 in a significant manner.

#### 3.5.3. Caspase 9

**Figure 8.**
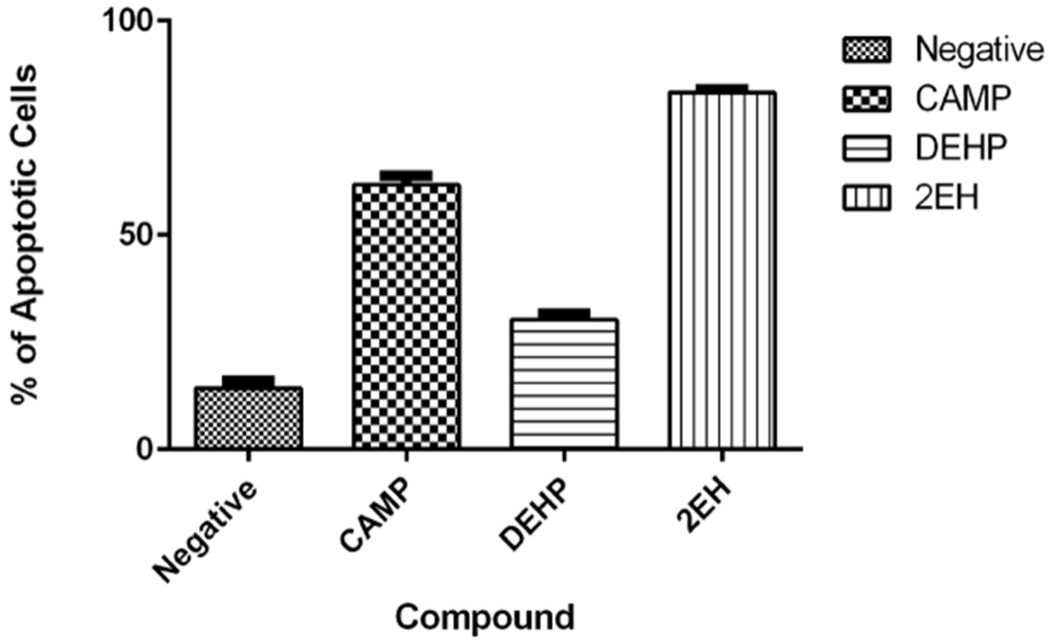
Caspase 9 activation. The negative control presented a low 14.5% of active caspase 9. Camptothecin samples averaged 62% and the phthalate treated samples also presented high activation of caspase 9 with an average 30% for DEHP and 83.25% for 2-EH.

Caspase 9 activation was significantly present on all treated samples only the negative control presented a low activation 14.5% of caspase 9. Camptothecin samples averaged 62% and the phthalate samples also presented high activation of caspase 9 with an average 30% for DEHP and 83.25% for 2-EH (Figure 8).

#### 3.5.4. Cell Cycle

**Figure 9.**
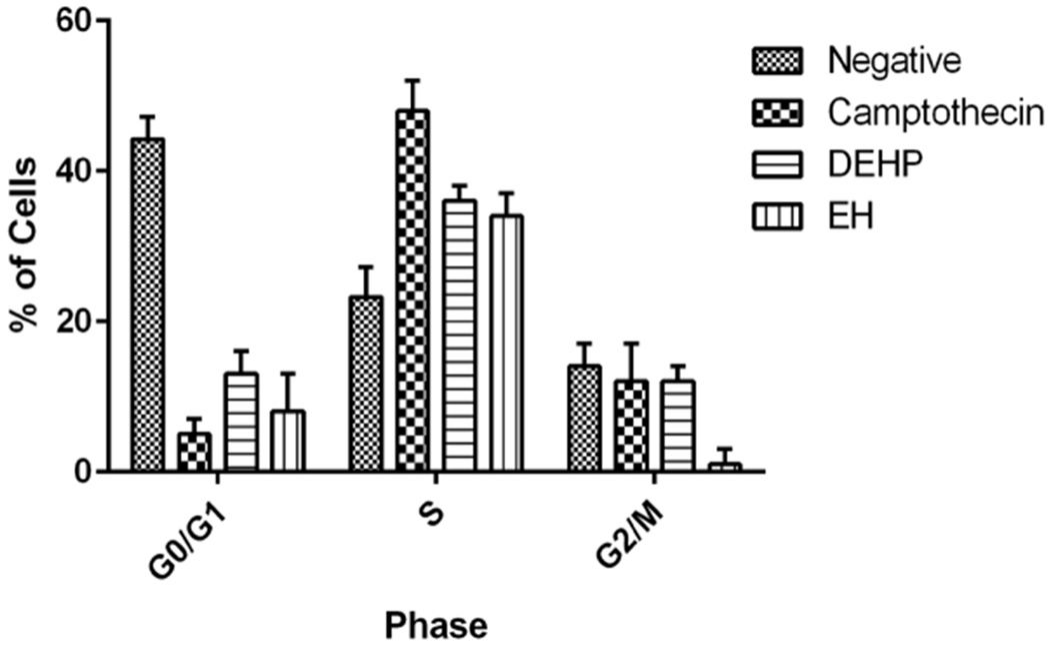
Cell cycle arrest. All the treated samples show some level of arrest at the S (synthesis) phase of the cell cycle. Camptothecin presents 48.0% of cells arrested at the S phase. DEHP and 2-EH presented 36.0% and 34.0% of cells arrested at the S phase respectively.

This analysis revealed that all the treated samples suggest cell cycle arrest at the S (synthesis) phase (Figure 9). The camptothecin positive control, a compound known to alter the cell cycle [22] presents a 48.0% of cells arrested at the S phase. DEHP and 2-EH presented 36.0% and 34.0% of cells arrested at the S phase respectively. These results confirm similar findings previously reported in which DEHP exposed cells presented S phase cell cycle arrest although no mechanistic explanation is know with certainty [23].

#### 3.5.5. Autophagy

**Figure 10.**
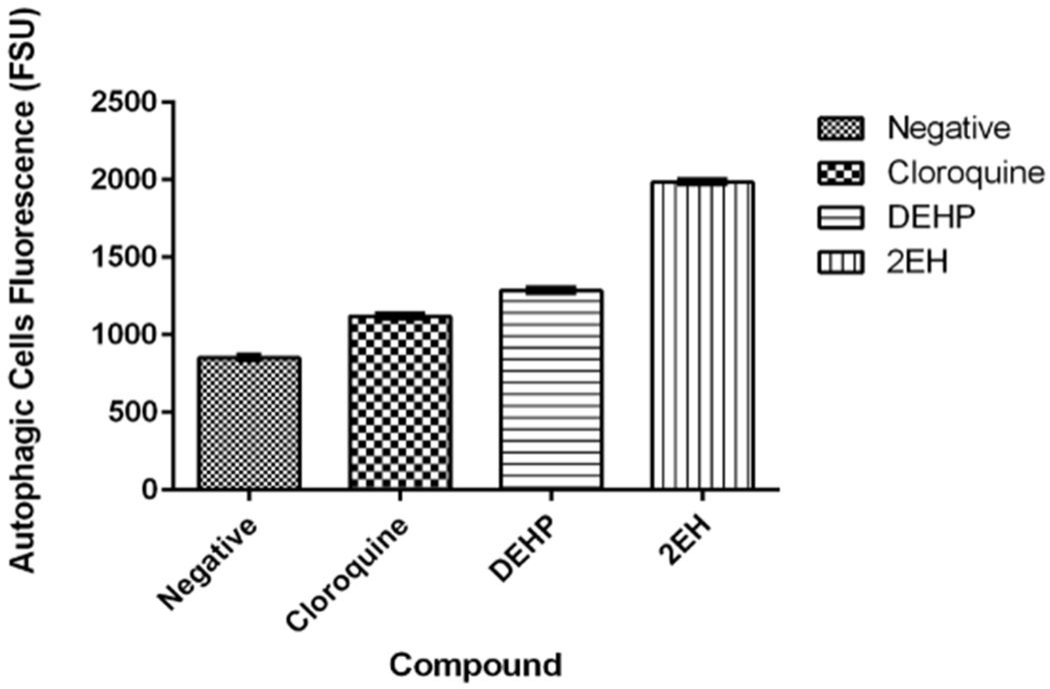
Autophagy activation. The negative control presented the lowest average FSU value of 852. Samples exposed to the test compounds presented statistically significant differences (*p* < 0.05) when compared to the negative control. Chloroquine averaged 1120 FSU while the DEHP and 2-EH averaged 1287 and 1984 FSU respectively.

Autophagy was detected in all the exposed samples. The negative control presented an average FSU value of 852 was the lowest one obtained. Samples exposed to the test compounds presented statistically significant differences (*p*< 0.05) when comparing them to the negative control. Chloroquine control averaged 1120 FSU while the DEHP and 2-EH averaged 1287 and 1984 FSU respectively (Figure 10).

#### 3.5.6. Cathepsin B

**Figure 11.**
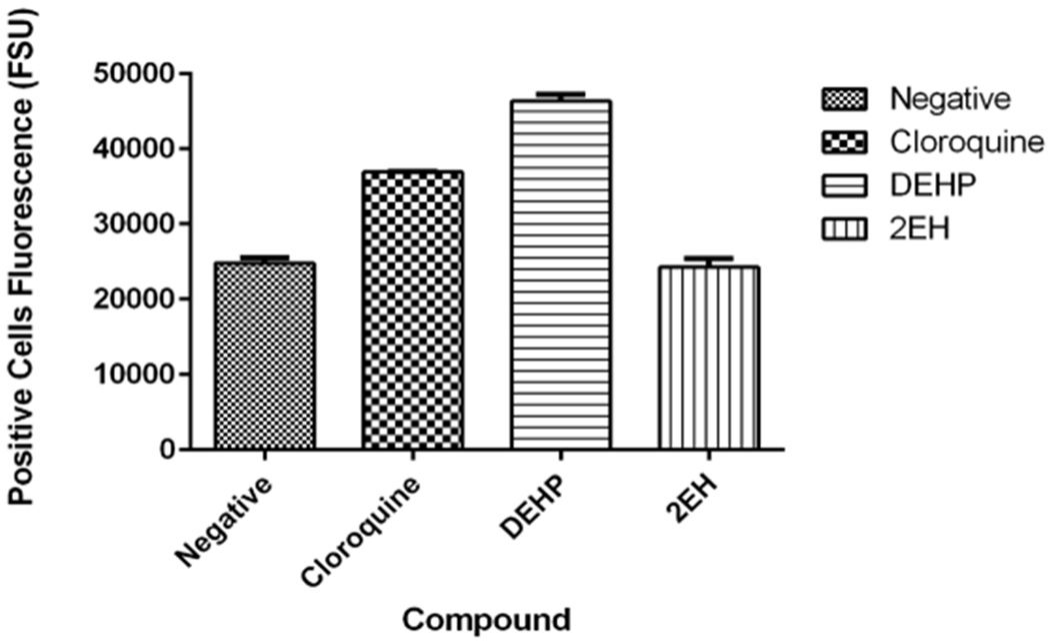
Detection of cathepsin B. Negative sample presented an average of 24,810.3 FSU and the 2-EH sample an average of 24,278.0 FSU. Positive control chloroquine samples presented an average of 36,947.5 FSU and the DEHP samples 46,342.2 FSU. Of the phthalate compounds, only DEHP was positive for cathepsin B staining.

Detection of cathepsins presented unexpected results, the negative control and 2-EH did not present any statistically significant (*p* < 0.05) results (Figure 11). The negative sample presented an average of 24,810.3 FSU and the 2-EH sample an average of 24,278.0 FSU. Control chloroquine samples presented an average of 36,947.5 FSU and the DEHP samples 46,342.2 FSU. In this essay, we can see that the DEHP shows an increase in cathepsin B while the 2-EH does not show a significant increase.

## 4. Discussion

The ubiquitous nature of phthalates in the modern world has caused an increase in the study of their biological interactions and potential health effects. Our previous work [10] presented the effects of MEHP and DEHP on TK6 lymphoblast cells. This current work expands on the previous findings by studying 2-EH a downstream metabolite of DEHP. Our results presented some differences in cell death hallmarks between DEHP and 2-EH. Our data shows that 2-EH is more toxic after 24-hour exposure on the TK6 lymphocytes. The GI_50_ dose of 4.4 μM shows higher 2-EH toxicity when comparing to DEHP with a 76 μM dose at 24 hours. Both compounds cause cell death however, some of the cell death hallmarks differ between DEHP and 2-EH.

Both compounds presented the traditional apoptosis hallmark of PS migration in the annexin V staining although at lower levels in DEHP than 2-EH. Additionally, both compounds presented mitochondrial membrane depolarization an event which has been documented in our previous work for MEHP and DEHP and by other researchers [10] [23]. It has been shown that mitochondria with collapsed ΔΨ_*m*_ are removed by autophagy [24]. Still, other research has proposed that degraded mitochondria induced by cathepsin B can lead to apoptosis [23]. In our results, 2-EH did not present significant levels of cathepsin B but was positive for the Premo autophagy assay which detects the LC3B protein found in lysosomes. DEHP presented signaling caspase activation (CASP 8 and 9) but in lower levels when compared to 2-EH which presented very high levels of active caspases. In terms of effector caspase (CASP 3) results presented some differences in activation DEHP presented some activation but not in a significant manner in contrast to 2-EH that did activate CASP 3 significantly. These CASP 3 results correlate with the observed DNA fragmentation results which presented low, albeit not significant results for DEHP while 2-EH results presented a high percentage (75.3%) of the cell with fragmented DNA. It is well understood that CASP 3 activation degrades DNA protein kinases, cytoskeletal proteins involved in chromatin condensation and nuclear fragmentation [25] [26]. Caspase activation during apoptosis can be a complex process because of the interplay that occurs between the different activation of extrinsic and intrinsic caspases [27]. This might explain the messenger caspase activation and variation in effector caspase observed in our results in which we observed CASP 8 and 9 activation but not significant CASP 3 in DEHP and all active caspases in 2-EH. It has been suggested by some authors that in certain cases conditions may arise in which an autophagic response is followed by apoptosis [28]. The cell cycle results reveal arrest at the S phase for both test compounds. Previous studies have proposed that the increase of cells in the S phase can be due to arrest in this phase or acceleration of the transition from G1 to S or both [29] [30] [31].

## 5. Conclusion

Based on our results, 2-EH shows activation of several different cell death events when comparing it to DEHP. The phthalate 2-EH presented higher toxicity with significant activation of caspases, alterations in mitochondrial potential, cell cycle arrest in S phase, the presence of autophagic lysosomes but no cathepsin B activation. Due to the interplay between the autophagic and apoptotic cell death events, further studies are needed to determine the mechanistic details and signaling events. The activation the majority of our tested cell death parameters by 2-EH and its higher toxicity warrants further mechanistic studies; its implications in public health and environmental impacts for these emerging contaminants.

## Figures and Tables

**Table 1.** Growth inhibition 50% after 24-hour exposure in TK6 cells. 2-EH presented the highest toxicity of 4.4 μM.

Compound	GI_50_ Dose
DEHP	76 μM
2-EH	4.4 μM

## References

[1] Fiore M, Conti GO, Caltabiano R, Buffone A, Zuccarello P, Cormaci L, Cannizzaro M and Ferrante M (2019) Role of Emerging Environmental Risk Factors in Thyroid Cancer: A Brief Review. International Journal of Environmental Research and Public Health, 16, 1185 10.3390/ijerph16071185 PMC6480006 30986998

[2] Bornehag CG, Lundgren B, Weschler CJ, Sigsgaard T, Hagerhed-Engman L and Sundell J (2005) Phthalates in Indoor Dust and Their Association with Building Characteristics. Environmental Health Perspectives, 113, 1399–1404. 10.1289/ehp.7809 16203254 PMC1281287

[3] Hauser R and Calafat AM (2005) Phthalates and Human Health. Occupational and Environmental Medicine, 62, 806–818. 10.1136/oem.2004.017590 16234408 PMC1740925

[4] Muscogiuri G and Colao A (2017) Phtalates: New Cardiovascular Health Disruptors? Archives of Toxicology, 91, 1513–1517. 10.1007/s00204-016-1780-1 27358237

[5] Benjamin S, Masai E, Kamimura N, Takahashi K, Anderson RC and Faisal PA (2017) Phthalates Impact Human Health: Epidemiological Evidences and Plausible Mechanism of Action. Journal of Hazardous Materials, 340, 360–383. 10.1016/j.jhazmat.2017.06.036 28800814

[6] Rowdhwal S and Chen J (2018) Toxic Effects of Di-2-Ethylhexyl Phthalate: An Overview. BioMed Research International, 2018, Article ID: 1750368 10.1155/2018/1750368 PMC5842715 29682520

[7] Quintana-Belmares RO, Krais AM, Esfahani BK, Rosas-Pérez I, Mucs D, López-Marure R, Bergman A and Alfaro-Moreno E (2018) Phthalate Esters on Urban Airborne Particles: Levels in PM10 and PM2.5 from Mexico City and Theoretical Assessment of Lung Exposure. Environmental Research, 161, 439–445. 10.1016/j.envres.2017.11.039 29216490

[8] Roslev P, Madsen PL, Thyme JB and Henriksen K (1998) Degradation of Phthalate and Di-(2-Ethylhexyl)phthalate by Indigenous and Inoculated Microorganisms in Sludge-Amended Soil. Applied and Environmental Microbiology, 64, 4711–4719. 9835553 10.1128/aem.64.12.4711-4719.1998 PMC90913

[9] Ito Y, Kamijima M, Hasegawa C, Tagawa M, Kawai T, Miyake M, Hayashi Y, Naito H and Nakajima T (2013) Species and Inter-Individual Differences in Metabolic Capacity of di(2-ethylhexyl)phthalate (DEHP) between Human and Mouse Livers. Environmental Health and Preventive Medicine, 19, 117–125. 10.1007/s12199-013-0362-6 24078404 PMC3944035

[10] Nalli S, Horn OJ, Grochowalski AR, Cooper DG and Nicell JA (2006) Origin of 2-Ethylhexanol as a VOC. Environmental Pollution, 140, 181–185. 10.1016/j.envpol.2005.06.018 16125828

[11] Rosado-Berrios CA, Vélez C and Zayas B (2011) Mitochondrial Permeability and Toxicity of Diethylhexyl and Monoethylhexyl Phthalates on TK6 Human Lymphoblasts Cells. Toxicology in Vitro. An International Journal Published in Association with BIBRA, 25, 2010–2016. 10.1016/j.tiv.2011.08.001 21864672 PMC3217166

[12] Crowley LC, Marfell BJ, Scott AP and Waterhouse NJ (2016) Quantitation of Apoptosis and Necrosis by Annexin V Binding, Propidium Iodide Uptake, and Flow Cytometry. Cold Spring Harbor Protocols, 2016. 10.1101/pdb.prot087288 27803250

[13] Thornton C and Hagberg H (2015) Role of Mitochondria in Apoptotic and Necroptotic Cell Death in the Developing Brain. Clinica Chimica Acta: International Journal of Clinical Chemistry, 451, 35–38. 10.1016/j.cca.2015.01.026 25661091 PMC4661434

[14] Pfeffer CM and Singh A (2018) Apoptosis: A Target for Anticancer Therapy. International Journal of Molecular Sciences, 19, 448 10.3390/ijms19020448 PMC5855670 29393886

[15] Errami Y, Naura AS, Kim H, Ju J, Suzuki Y, El-Bahrawy AH, Ghonim MA, Hemeida RA, Mansy MS, Zhang J, Xu M, Smulson ME, Brim H and Boulares AH (2013) Apoptotic DNA Fragmentation May Be a Cooperative Activity between Caspase-Activated Deoxyribonuclease and the Poly(ADP-ribose) Polymerase-Regulated DNAS1L3, an Endoplasmic Reticulum-Localized Endonuclease That Translocates to the Nucleus during Apoptosis. The Journal of Biological Chemistry, 288, 3460–3468. 10.1074/ibc.M112.423061 23229555 PMC3561564

[16] Moonen L, D’Haese PC and Vervaet BA (2018) Epithelial Cell Cycle Behaviour in the Injured Kidney. International Journal of Molecular Sciences, 19, 2038 10.3390/ijms19072038 PMC6073451 30011818

[17] Mishra P, Ammanathan V and Manjithaya R (2018) Chemical Biology Strategies to Study Autophagy. Frontiers in Cell and Developmental Biology, 6, 160 10.3389/fcell.2018.00160 30538986 PMC6277461

[18] Li X, Li J, Zhang Y and Zhou Y (2016) Di-n-butyl Phthalate Induced Hypospadias Relates to Autophagy in Genital Tubercle via the PI3K/Akt/mTOR Pathway. Journal of Occupational Health, 59, 8–16. 10.1539/joh.16-0089-OA 27885243 PMC5388616

[19] Mizushima N, Yoshimori T and Levine B (2010) Methods in Mammalian Autophagy Research. Cell, 140, 313–326. 10.1016/j.cell.2010.01.028 20144757 PMC2852113

[20] Liu N, Jiang L, Sun X, Yao X, Zhai X, Liu X, Wu X, Bai Y, Wang S and Yang G (2017) Mono-(2-ethylhexyl) Phthalate Induced ROS-Dependent Autophagic Cell Death in Human Vascular Endothelial Cells. Toxicology in Vitro, 44, 49–56. 10.1016/j.tiv.2017.06.024 28655635

[21] Siklos M, BenAissa M and Thatcher GR (2015) Cysteine Proteases as Therapeutic Targets: Does Selectivity Matter? A Systematic Review of Calpain and Cathepsin Inhibitors. Acta Pharmaceutica Sinica B, 5, 506–519. 10.1016/j.apsb.2015.08.001 26713267 PMC4675809

[22] Uchiyama Y (2001) Autophagic Cell Death and Its Execution by Lysosomal Cathepsins. Archives of Histology and Cytology, 64, 233–246. 10.1679/aohc.64.233 11575420

[23] Wang X, Tanaka M, Krstin S, Peixoto HS, Moura CCM and Wink M (2016) Cytoskeletal Interference—A New Mode of Action for the Anticancer Drugs Camptothecin and Topotecan. European Journal of Pharmacology, 789, 265–274. 10.1016/j.ejphar.2016.07.044 27474470

[24] Ma Y, Guo Y, Wu S, Lv Z, Zhang Q, Xie X and Ke Y (2017) Analysis of Toxicity Effects of Di-(2-ethylhexyl) Phthalate Exposure on Human Bronchial Epithelial 16HBE Cells. Cytotechnology, 70, 119–128. 10.1007/s10616-017-0111-6 28689280 PMC5809640

[25] Wu X, Jiang L, Sun X, Yao X, Bai Y, Liu X, Liu N, Zhai X, Wang S and Yang G (2017) Mono(2-ethylhexyl) Phthalate Induces Autophagy-Dependent Apoptosis through Lysosomal-Mitochondrial Axis in Human Endothelial Cells. Food and Chemical Toxicology, 106, 273–282. 10.1016/j.fct.2017.05.069 28579546

[26] Kim I and Lemasters JJ (2011) Mitophagy Selectively Degrades Individual Damaged Mitochondria after Photoirradiation. Antioxidants & Redox Signaling, 14, 1919–1928. 10.1089/ars.2010.3768 21126216 PMC3078512

[27] Jafri A, Siddiqui S, Rais J, Ahmad MS, Kumar S, Jafar T, Arshad M, (2019) Induction of Apoptosis by Piperine in Human Cervical Adenocarcinoma via ROS Mediated Mitochondrial Pathway and Caspase-3 Activation. EXCLI Journal, 18, 154–164. 31217779 10.17179/excli2018-1928 PMC6558508

[28] Erekat NS (2018) Apoptosis and Its Role in Parkinson’s Disease In: Stoker TB and Greenland JC, Eds., Parkinson’s Disease: Pathogenesis and Clinical Aspects, Codon Publications, Brisbane, Chapter 4.

[29] Mariño G, Niso-Santano M, Baehrecke EH and Kroemer G (2014) Self-Consumption: The Interplay of Autophagy and Apoptosis. Nature Reviews Molecular Cell Biology, 15, 81–94. 10.1038/nrm3735 24401948 PMC3970201

[30] Maiuri MC, Zalckvar E, Kimchi A and Kroemer G (2007) Self-Eating and Self-Killing: Crosstalk between Autophagy and Apoptosis. Nature Reviews Molecular Cell Biology, 8, 741–752. 10.1038/nrm2239 17717517

[31] Ma Y, Guo Y, Wu S, Lv Z, Zhang Q, Xie X and Ke Y (2018) Analysis of Toxicity Effects of Di-(2-ethylhexyl) Phthalate Exposure on Human Bronchial Epithelial 16HBE Cells. Cytotechnology, 70, 119–128. 10.1007/s10616-017-0111-6 28689280 PMC5809640

